# Isolation and Identification of Long Non-Coding RNAs in Exosomes Derived from the Serum of Colorectal Carcinoma Patients

**DOI:** 10.3390/biology10090918

**Published:** 2021-09-15

**Authors:** Chin Tat Ng, Shamin Azwar, Wai Kien Yip, Siti Yazmin Zahari Sham, Mohd Faisal Jabar, Norren Haneezah Sahak, Norhafizah Mohtarrudin, Heng Fong Seow

**Affiliations:** 1 Department of Pathology, Faculty of Medicine and Health Sciences, Universiti Putra Malaysia, UPM Serdang 43400, Selangor, Malaysia; jackngchintat@gmail.com (C.T.N.); shamin.azwar@gmail.com (S.A.); lab1@viascientia.com.my (W.K.Y.); sitiyazmin@upm.edu.my (S.Y.Z.S.); shf@upm.edu.my (H.F.S.); 2 Department of Surgery, Faculty of Medicine and Health Sciences, Universiti Putra Malaysia, UPM Serdang 43400, Selangor, Malaysia; faisal@upm.edu.my; 3 Department of Pathology, Hospital Serdang, Jalan Puchong, Kajang 43000, Selangor, Malaysia; hsdg@moh.gov.my

**Keywords:** lncRNA, colorectal cancer, exosomes, H19, LINC00152

## Abstract

**Simple Summary:**

Treatment regimens for patients with advanced disease are limited and the mortality rate is high in these patients. A better understanding on pathogenesis and progression of cancer is critical for the development of new treatment strategies. In colorectal cancer (CRC), exosomes (secreted vesicles from cells) and long non-coding RNAs (lncRNAs) have been shown to play significant roles in disease development and progression. Long non-coding RNAs (lncRNAs) are present in the exosomes of serum and their profiles may potentially be useful as novel biomarkers for CRC patients and may provide a new insight in the pathogenesis and progression of CRC. Here, we compared the expression profiles of exosomal lncRNAs between non-cancer individuals and patients with colorectal carcinoma. The relative expression level of LINC00152 was found to be significantly lower in exosomes from sera of CRC patients as compared to non-cancer individuals whereas lncRNA H19 was significantly up-regulated in advanced-stages (stage III and IV) of CRC as compared to early-stages (stage I and II). Our data suggest that LINC00152 and H19 may play important roles in pathogenesis and progression of CRC.

**Abstract:**

Long non-coding RNAs (lncRNAs) are non-coding RNAs consisting of more than 200 nucleotides in length. LncRNAs present in exosomes may play a critical role in the cellular processes involved in cancer pathogenesis and progression including proliferation, invasion, and migration of tumor cells. This paper aims to identify the differential expression of exosomal lncRNAs derived from the sera of non-cancer individuals and patients diagnosed with colorectal carcinoma. These differentially-expressed exosomal serum lncRNAs may provide an insight into the pathogenesis and progression of colorectal cancer (CRC). Serum exosomes and exosomes from SW480-7 cell culture supernatants were isolated and viewed by transmission electron microscope (TEM). The particle size distribution and protein markers of exosomes derived from SW480-7 were further analyzed using the Zetasizer Nano S instrument and western blotting technique. TEM showed that exosomes derived from serum and SW480-7 cells were round vesicles with sizes ranging from 50–200 nm. The exosomes derived from SW480-7 had an average diameter of 274.6 nm and contained the exosomal protein, ALIX/PDCD6IP. In our clinical studies, six lncRNAs, namely GAS5, H19, LINC00152, SNHG16, RMRP, and ZFAS1 were detected in the exosomes from sera of 18 CRC patients. Among these six lncRNAs, the expression level of LINC00152 was found to be significantly lower in CRC patients as compared to non-cancer individuals (*p* = 0.04) while lncRNA H19 was significantly up-regulated in advanced-stages (stage III and IV) of CRC (*p* = 0.04) as compared to early-stages (stage I and II). In conclusion, the detection of lower LINC00152 in exosomes of sera from CRC patients versus non-cancer individuals and H19 upregulation in advanced stages suggests that they may play important roles in pathogenesis and progression of CRC.

## 1. Introduction

Colorectal cancer (CRC) is the third most common cancer worldwide [1] that contributes to a high mortality rate, despite decades of research and improvement in clinical management [2]. In 2018, CRC has caused an estimated 880,792 deaths (9.2% of total cancer death), and about 1.85 million new cases (10.2% of total new cases) in 185 countries [3]. Recent literature in the pathogenesis of CRC has mainly focused on the involvement of an increasing number of non-coding ribonucleic acids (ncRNAs) via next-generation sequencing and transcriptome analysis [4,5]. These ncRNAs encompass a variety of subclasses that include short regulatory ncRNAs such as miRNAs, siRNAs, and piRNAs, as well as the long non-coding RNAs (lncRNAs).

LncRNAs are transcripts of more than 200 nucleotides [6] in length with no or limited protein-coding potential and have recently been found in significant amounts inside exosomes. In the past, lncRNAs were described as “transcriptional noise” [7,8]. However, recent studies have demonstrated that lncRNAs play important roles in central cellular processes such as in epigenetic modulation, transcription, and translation. When compared with protein-coding genes, lncRNAs are tissue-specific although expressed at low levels [7,9]. In CRC, lncRNAs have been shown to play significant roles in carcinogenesis via various cellular processes such as in cellular proliferation [10,11,12], apoptosis [13], migration [10,14], invasion, and metastasis [10]. Exosomes are extracellular vesicles ranging between 20–120 nm in-size and carry a significant amount of nucleic acids that include lncRNAs. These vesicles are secreted into the extracellular space by various cells including cancer cells. Tumor-derived exosomes can trigger the angiogenic process and enhance tumor metastasis. Exosomes can be used as a liquid biomarker for various cancer diagnoses as they mirror the contents of the original parental tumor cells [15,16]. Current screening tests are deemed inadequate and factors such as false positive or negative results, laborious procedures, and expensive molecular testing remain as major obstacles for the early detection of CRC. Hence, there is a dire need for the development of a diagnostic test for cancer detection. Exosomes carrying biomarkers specific to the origin of cancer cells [15] are present in serum and their profiles may potentially be useful as novel biomarkers for CRC patients and may emerge as a new diagnostic approach as well as provide information on the pathogenesis and progression of the disease.

Circulating exosomal markers in serum have yet to be developed for the detection of CRC. Recently circulating miRNAs or exosomal miRNAs have been recognized as promising biomarkers for ovarian cancer [17], lung cancer [18], colon cancer [19,20,21], prostate cancer [22], and breast cancer [23]. It is likely that circulating exosomes in body fluids containing lncRNAs may present new, relatively non-invasive cancer biomarkers. The results will enable the development of diagnostic and prognostic tests [5] for non-invasive and early detection of CRC. Moreover, there is an increasing interest in the role of exosomal lncRNAs in the pathogenesis and progression of CRC [24,25]. Hence, in our study, we aim to detect the exosomal lncRNAs in sera from non-cancer individuals and patients with colorectal carcinoma, examine the lncRNA expression profile and identify the differentially expressed exosomal lncRNAs related to colorectal carcinoma. We hypothesized that there are significant differences in the expression of exosomal lncRNAs between apparently healthy individuals and patients with colorectal carcinoma.

## 2. Materials and Methods

### 2.1. Ethics Approval

Serum samples were collected from Hospital Serdang with the informed consent from patients, and the study was approved by the Malaysian Ministry of Health Research Ethics Committee (NMRR-14-1927-23392). All experiments were conducted according to the institutional ethical guidelines.

### 2.2. Serum Preparation and Storage

Whole blood was collected in blood collection tubes (BD Vacutainer^®^ SSTTM). Tubes were left at room temperature (15–25 °C) for at least 30 min, before being centrifuged at 1000× *g* for 15 min. The serum-containing upper phase was then transferred to a new tube, filtered with a 0.22 μm polyethersulfone (PES) filter, and aliquoted into microcentrifuge tubes for storage at −80 °C. All blood samples were taken from newly-diagnosed patients via colonoscopy prior to surgery, and that these patients have not undergone any treatment such as neoadjuvant chemotherapy or radiation.

### 2.3. Vesicle Isolation from Serum Sample

Exosomes were isolated using the ExoRNeasy Serum/Plasma Midi Kit (Qiagen GmBH, Hilden, Germany) according to the manufacturer’s protocol. Briefly, Buffer XBP was added into the serum sample in a 1:1 ratio. The mixture was then gently inverted five times before left at room temperature for 5 min. It was then pipetted into an exoEasy spin column and centrifuged at 500× *g*. The flow-through was discarded and the column was placed back into the same collection tube. To wash the membrane, 3.5 mL Buffer XWP was added into the spin column and centrifuged at 3000× *g* for 5 min. Flow-through was again discarded and the column was placed to a fresh collection tube. For elution, 100 μL of Buffer XE was added directly onto the membrane and centrifuged at 3000× *g* to collect the purified exosomes.

### 2.4. Cell Culture and Vesicle Isolation from Culture Supernatants of SW480-7

An invasive subpopulation of colon cancer cell line SW480, namely, SW480-7 was previously-established via seven sequential passages of cells through the Matrigel-coated transwells [26]. SW480-7 was cultured in RPMI 1640 medium supplemented with 10% FBS 100 U/mL penicillin, 100 μg/mL streptomycin, and 2 g/L sodium bicarbonate (Gibco, Invitrogen Corp., Carlsbad, CA, USA). Once the cells grew to subconfluency (approximately 75%), the old medium was discarded and the flask was washed 2–3 times with Dulbecco’s phosphate-buffered saline (D-PBS) solution. The SW480-7 cells were then cultured in RPMI 1640 medium supplemented with exosome-depleted FBS (Gibco, Thermo Fisher Scientific, Inc., Waltham, MA, USA), 100 U/mL penicillin, 100 μg/mL streptomycin, and 2 g/L sodium bicarbonate for 48 h. After 48 h, culture supernatants were transferred to a 15 mL conical tube, centrifuged for 15 min at 3000× *g* and at 4 °C to remove cellular fragments and cell debris. Cleared supernatants were then transferred to a new 50 mL conical tube. Exosomes derived from SW480-7 were isolated using exoEasy Maxi Kit (Qiagen, GmBH, Hilden, Germany) according to the manufacturer’s protocol. The eluate containing exosomes was then transferred to 1.5 mL protein low binding tubes. Purified exosomes were stored at −20 °C for short term storage, and at −80 °C for longer storage.

### 2.5. Exosomal Protein Determination

Lysate mixture was prepared by mixing 50 µL of exosomal eluate with an equal volume of 2× radioimmunoprecipitation assays (RIPA) lysis buffer. Twenty-five µL of the lysate mixture was used to quantify exosomal protein content via the Micro BCA Protein assay kit (Cat. No. 23227, Thermo Fisher Scientific) according to the manufacturer’s instructions with BSA as standard. Samples and standards were mixed with the working reagent and incubated for 30 min at 37 °C. Absorbances readings were measured at 570 nm wavelength.

### 2.6. Detection of ALIX/PDCD6IP in Exosomes

To extract proteins from SW480-7 cells, adherent cells in the petri dish were lysed with 100 µL of 1× SDS sample buffer (62.5 mM Tris-HCl (pH 6.8), 2% *w/v* SDS, 10% glycerol, 100 mM DTT). These adherent cells in lysis buffer were scraped off from the culture dish using a plastic cell scraper and homogenized by passing the suspension through a 27-gauge needle with 1 mL sterile plastic syringe for several times. The homogenized cells were transferred into a microcentrifuge tube and heated at 95–100 °C for 8 min, pulsed centrifuged, and an equal volume of 2× sample buffer was added. Then, the tube was shaken vigorously to homogenize the sample. The sample was boiled at 95 °C for 5 min and immediately cooled at room temperature. The protein sample was then kept at −20 °C.

Proteins were loaded for SDS-polyacrylamide (SDS-PAGE) gel electrophoresis using the Mini-PROTEAN^®^ Tetra Cell electrophoresis system (Bio-Rad Laboratories, Hercules, CA, USA), electrophoresed at 120 V for 1 h and the resolved proteins were transferred to 0.45 µM PVDF membranes (Pierce, Thermo-Scientific, Waltham, MA, USA) for 2 h at 100 V. The membranes were washed with TBS/T for 5 min and subsequently blocked with blocking buffer (5% BSA in 0.1% Tween-20 TBS) for 1 h. Next, the membrane was incubated with the primary antibody, ALIX/PDCD6IP (dilution: 1:2000: Cat. No. 92880, Cell Signaling) with gentle agitation overnight at 4 °C. After 24 h, the membranes were then washed three times (5 min each) with 0.1% Tween-20 TBS, followed by incubation with anti-mouse antibody conjugated with horseradish peroxidase (1:2000) for 1 h at room temperature with gentle agitation. The membranes were washed two times (5 min each) with 0.1% Tween-20 TBS and one time with TBS before the detection of protein bands. Visualization of protein bands was conducted by adding 150 µL of chemiluminescent substrate (SuperSignal^®^ West Femto Maximum Sensitivity Substrate; Thermo Fisher Scientific, Rockford, IL, USA) solution to the membranes for 5 min. After 5 min, the membrane covered with a transparent plastic and protein bands were visualized with the Fluorchem 5500 imaging system (Alpha Innotech Corporation, San Leandro, CA, USA).

### 2.7. Size Distribution Analysis

The size of exosomes derived from SW480-7 cell culture supernatants was determined by photon correlation spectroscopy technique using the Zetasizer Nano S instrument (Malvern Instrument, Malvern, UK). Twenty µL of exosomes (143 µg/mL) derived from SW480-7 were diluted in 1 mL of ddH_2_O. The particle size and size distribution were measured as cumulant (z-average) size and polydispersity index (PDI).

### 2.8. Determination of Exosome Morphology by Transmission Electron Microscopy (TEM)

The morphology of exosomes isolated from serum and SW480-7 cell culture supernatants were evaluated by TEM. Exosomal samples were stored at −80 °C and thawed at room temperature. After thawing, a drop (10 µL) of the exosomes was placed on a sheet of parafilm. A carbon-coated copper grid (200 mesh and coated by formvar carbon film) was covered by a drop of exosomes for 5 min. The excess liquid was absorbed by a clean filter paper. The grid was incubated with a drop of 1% uranyl acetate at room temperature for 3–5 min. The excess uranyl acetate was absorbed by filter paper. Then, the copper grid was washed twice with pre-filtered sterile distilled water and incubated at room temperature. The samples were kept overnight in a 1.5 mL tube and then viewed with a Hitachi 7100 transmission electron microscope.

### 2.9. RNA Extraction

Total RNA was extracted from isolated exosomes with ExoRNeasy Serum/Plasma Midi Kit (Qiagen GmBH, Hilden, Germany) according to manufacturer instructions. The purity and quantity of RNA were detected by using the Nanodrop 1000 spectrophotometer (Thermo Fisher Scientific, Inc., Wilmington, DE, USA).

### 2.10. Reverse Transcription (RT)

RT of lncRNA was conducted with RT^2^ PreAMP cDNA Synthesis Kit (Qiagen GmBH, Hilden, Germany) and Ovation Pico WTA System V2 following the manufacturer’s protocol. The extracted RNA (4 µL) was treated with 1 µL of Buffer GE from the RT^2^ PreAMP cDNA Synthesis Kit. Half the volume of standard cDNA synthesis reagents was used. Preamplification of cDNA target templates was then conducted using RT^2^ PreAMP cDNA Synthesis Kit according to the manufacturer’s protocol.

### 2.11. Detection of lncRNA in Exosomes by RT-PCR

LncRNAs in exosomes were detected using RT^2^ lncRNA PCR Array, Human Cancer Pathway Finder (Cat. No. 330721 LAHS-002Z, Qiagen). After reverse transcription, real-time quantitative polymerase chain reaction (qPCR) was conducted using a real-time thermocycler (Eppendorf, Wesseling-Berzdorf, Germany) with the cycling condition as shown in Table 1.

### 2.12. Statistical Analysis

Data analysis was performed at Qiagen’s GeneGlobe Data Analysis Center, a web resource for real-time PCR analysis using the ∆∆Cq method. The student’s *t*-test was used to compare the normalized lncRNA expression levels and two-sided *p* < 0.05 was considered to be statistically significant.

## 3. Results

### 3.1. Characterization of Exosomes Derived from Serum Sample and SW480-7 Cell Line

**Figure 1:**
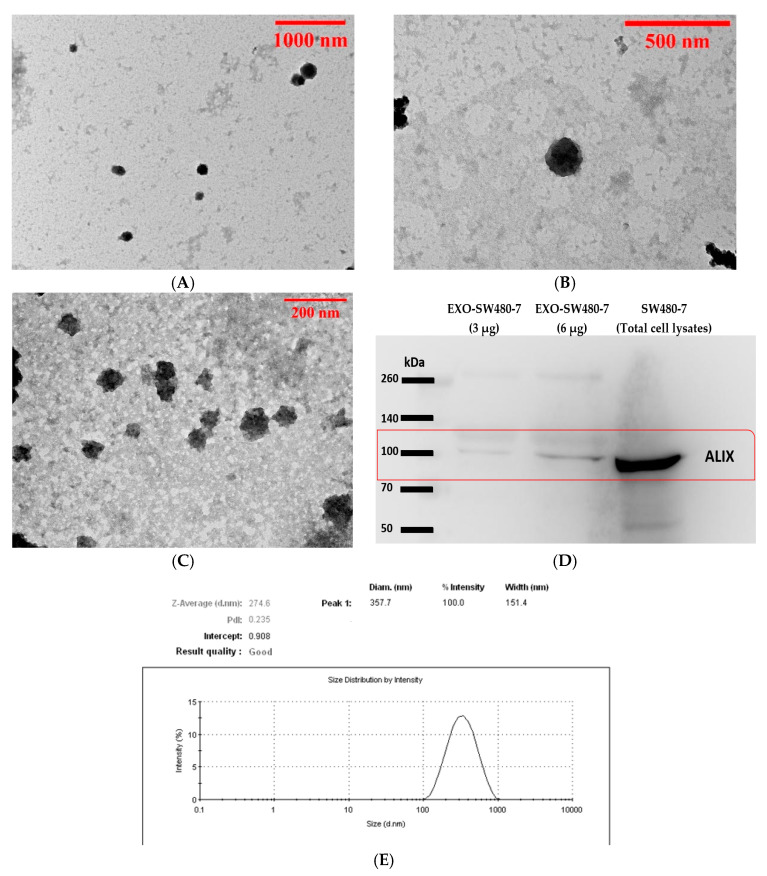
Characterization of exosomes. (**A**,**B**) Morphology of serum exosomes by transmission electron microscopy (TEM). (**C**) Morphology of exosomes derived from SW480-7. (**D**) Western blot analysis of exosomal protein PDCD6IP/ALIX derived from SW480-7. (**E**) The particle size distribution of exosomes derived from SW480-7 cell line.

The morphology of exosomes was characterized by TEM analysis. The exosomes derived from serum (Figure 1A,B) and SW480-7 (Figure 1C) were round-shaped in appearance. TEM analysis results showed that the size of the exosomes is less than 200 nm in diameter (Figure A–C). The size distribution of exosomes derived from cell culture supernatants of SW480-7 was determined by Zetasizer analysis. The average diameter of exosome-like vesicles isolated for SW480-7 was 274.6 nm (ranging from 105.7–955.4 nm) as shown in Figure 1E. To confirm that the exosome isolated contained proteins associated with exosomes, western blot analysis was performed. Two amounts of exosomes, 3 µg, and 6 µg were used in this experiment. As shown in Figure 1D, a band of approximately 100 kDa which is the expected size for ALIX/PDCD6IP was detected in exosomes derived from SW480-7 cells and whole cell lysate from SW480-7 (All western blot images captured at 1 to 5 min exposure time are available in Figure S1a–e). Due to the minuscule amount of serum-derived exosomes, Zetasizer and western blot analysis was not performed on exosomes from sera of CRC patients, but on exosomes isolated from SW480-7 cells instead.

### 3.2. Clinical and Pathological Characteristics

The clinical and pathological data of 18 CRC patients are detailed in Table 2. The patient population is composed of 9 males and 9 females. Ten patients were above 60 years old while 8 others were of below 60 years of age. According to TNM staging classification, there was one from stage I. Stages II, III both consisted of 7 samples, while stage IV consisted of 3 samples.

### 3.3. Detection of Exosomal lncRNAs Expression in CRC Patients and Non-Cancer Individuals

**Figure 2:**
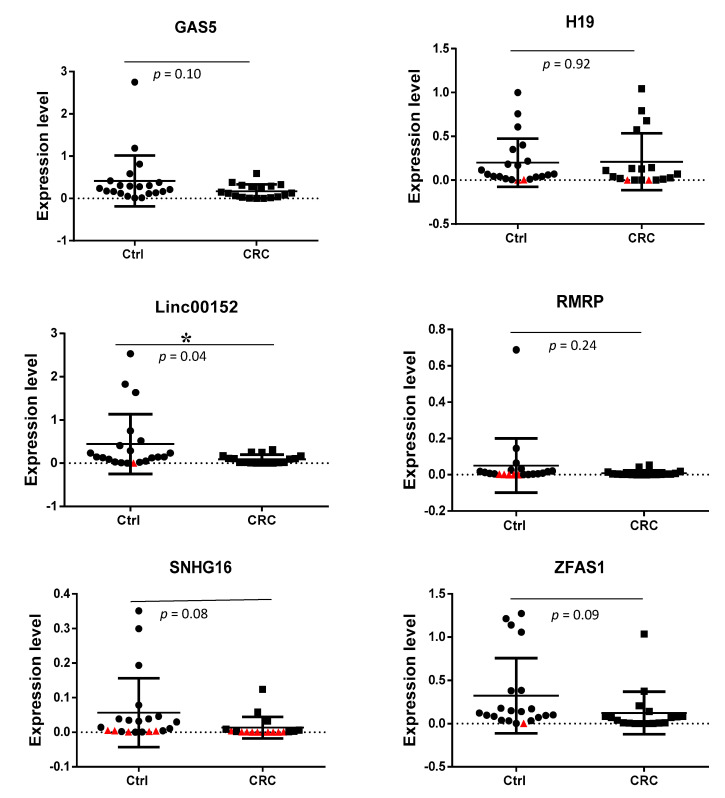
Comparison of lncRNAs expression level (2^(−ΔCt)^) in serum exosomes from CRC and non-cancer individuals (Ctrl). The levels of lncRNA expression have been normalized to the reference gene, B_2_M. The student’s *t*-test was used to compare the expression of six lncRNAs between non-cancer individuals and CRC patients, * indicates *p* < 0.05. Samples with Ct value above the cut-off value of 30 were considered as negative expression. Samples that could not be amplified were plotted as red triangles.

The expression levels of lncRNAs GAS5, H19, LINC00152, RMRP, SNHG16, and ZFAS1 were detected in serum-derived exosomes of CRC patients and non-cancer individuals via RT-qPCR (All lncRNAs expression level are available in Table S2). Table 3 and Figure 2 summarizes the data representing the level of lncRNA expression normalized to that of a reference gene, B_2_M. The student’s *t*-test was used to compare the expression of six lncRNAs between non-cancer individuals and CRC patients. Differences were considered to be statistically significant at *p* < 0.05. There was no significant difference in the expression of GAS5, H19, RMRP, SNHG16, and ZFAS1 between CRC patients and non-cancer individuals. However, the results show that LINC00152 was significantly down-regulated in serum-derived exosomes of CRC patients when compared to the non-cancer individuals (*p* < 0.05).

### 3.4. Detection of Exosomal lncRNAs Expression in Early and Advanced Stages of CRC Patients

**Figure 3:**
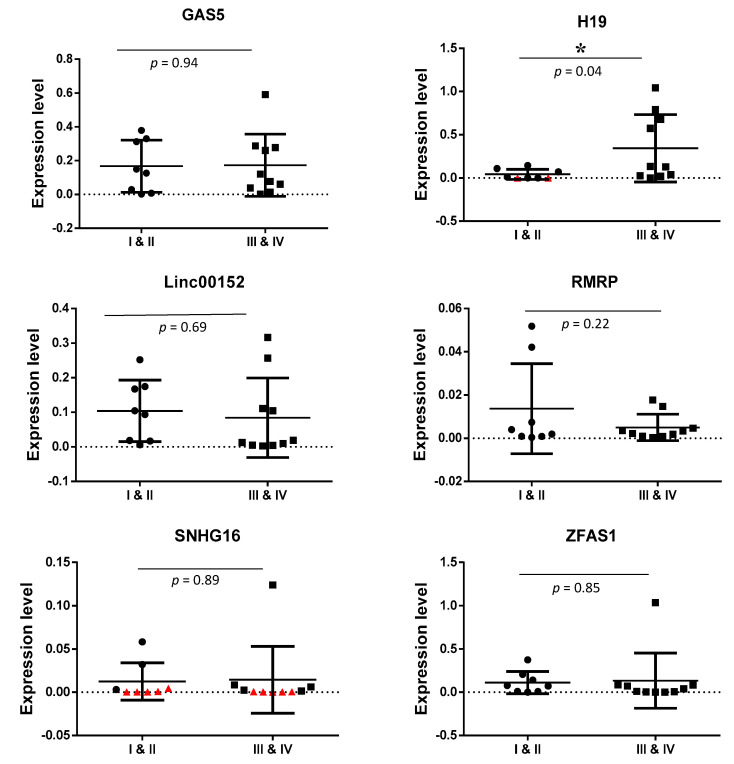
Comparison of lncRNAs expression level (2^(−ΔCt)^) in serum exosomes from early (I and II) and advanced (III and IV) stages of CRC patients. The levels of lncRNA expression have been normalized to the reference gene, B2M. Student’s *t*-test was used to compare the expression of six long non-coding RNAs between early-stages and advanced stages of CRC patients, * indicates *p* < 0.05. Samples that could not be amplified were plotted as red triangles.

The expression level of lncRNAs GAS5, H19, LINC00152, RMRP, SNHG16, and ZFAS1 were detected in exosomes from serum of early-stages and advanced stages of CRC patients by RT-qPCR (All lncRNAs expression level are available in Table S1). Table 4 and Figure 3 summarizes the data representing the level of lncRNA expression normalized to a reference gene, B_2_M. The student’s *t*-test was used to compare the expression of six lncRNAs between early-stages and advanced stages of CRC patients. Differences were considered to be statistically significant at *p* < 0.05. There was no significant difference in the expression level of GAS5, LINC00152, RMRP, SNHG16, and ZFAS1 between early-stages and advanced-stages of CRC patients. However, the result showed that H19 was significantly up-regulated in exosomes from the serum of advanced-stages of CRC patients compared with the early-stages (*p* < 0.05).

## 4. Discussion

In the present study, exosomes from serum and cell culture supernatants were isolated using the membrane affinity spin column method. Serum samples were filtered with a 0.22 μm PES filter to eliminate larger size vesicles before the isolation of exosomes. TEM shows that the size of the exosomes from serum and cell culture supernatant of SW480-7 is in the expected range and no cell debris or apoptotic bodies are found. This result is consistent with a previous study showing eluate from the spin column method has less granular background staining as compared with the ultracentrifugal isolation method [27]. Size distribution and western blot analysis of the exosomes derived from SW480-7 revealed the exosomes were within the expected size and harbored the presence of an exosomal protein marker, respectively.

Recently, the role of exosomes in cancer progression has gained great interest [28]. The role of exosomal lncRNAs in the early event of pre-metastatic niche formation has also become the focus of many researchers [29,30]. Exosomes secreted from tumor cells contain biomolecules such as protein, RNA (coding mRNA or non-coding RNA), DNA, and glycans. These biomolecules play important roles in the remodeling of the extracellular matrix, stimulating pro-tumorigenic processes, inducing immune suppression, and promoting cancer cell migration, and invasion [28,29,31]. Exosomal non-coding RNAs involved in pre-metastatic niche formation include exosomal miR-25-3p that promote angiogenesis via increased the permeability of vascular endothelial cells [31]. Although the role of exosomal microRNAs in pre-metastatic niche formation has been reported, however, a similar role by exosomal lncRNAs in CRC is still poorly understood.

In the current study, we investigated the differentially expressed exosomal lncRNAs from non-cancer individuals and patients with colorectal carcinoma. The expression level of exosomal lncRNAs from serum samples was detected using an RT^2^ lncRNA PCR Array. The cut-off Ct value was set at 30. Six genes from the array panel were considered as positive when their Ct values were lower than the cut-off value. These six lncRNAs found were GAS5, H19, RMRP, SNHG16, LINC00152, and ZFAS1and they were detected in more than 60% from CRC patients and non-cancer individuals. Surprisingly, in this current study, we found that oncogenic lncRNAs such as CCAT1, CCAT2, CRNDE, FTX, HOTAIR, HOTTIP, HULC, MALAT1, PCAT1, PVT1, and UCA1 were either not detected or were detected at a very low rate in both non-cancer individuals and CRC patients. In CRC, circulating exosomal lncRNAs such as CRNDE-h and BCAR4 from serum samples have been previously reported [24,32]. Other oncogenic and tumor suppressor lncRNAs such as TUS7 S and MEG3 were also not detected. It has been reported that the concentration, purity, and size of exosomes and types of exosomal RNAs are different due to the difference in the isolation technique used [33]. This could be the reason why the lncRNAs detected in previous studies are not detected in this study.

LINC00152 has been reported to function as a competitive endogenous RNA (ceRNA) by sponging tumor suppressor miRNAs such as miR-193a-3p [34], miR-632, and miR-185-3p [35] to promote proliferation and metastasis of CRC. Our study revealed that LINC00152 was significantly lower in the exosomes of serum from CRC patients (−3.23-fold, *p* = 0.04) as compared to that of non-cancer individuals. To the best of our knowledge, this is the first finding showing a lower level of LINC00152 in exosomes derived from serum of CRC patients. This result is consistent with a previous finding showing an abnormally low level in CRC tissues and cells [34]. In that same study, the low level of LINC00152 was found more frequently in CRC patients with advanced tumors (stage III and IV) which is also consistent with our current study [36].

H19 is a lncRNA that has a dual role as it can act both as an oncogene or tumor suppressor [37]. Functional investigations showed that knockdown of H19 resulted in inhibition of proliferation, migration, and invasiveness of CRC cells. H19 was reported to be up-regulated in cancerous tissues [38] and overexpression of H19 was related to distant metastasis and poor prognosis in patients with CRC [39]. In the current study, higher expression of H19 was found in the exosomes of sera from the advanced-stages of CRC patients as compared to the early stages of CRC patients. These results suggest that exosomal H19 acting as an oncogene has a role in disease progression as it is highly secreted in the advanced-stages of CRC patients as compared to early-stage CRC. Future studies using CRISPR/Cas-9 technology can be carried out to further understand the mechanism underlying the role of H19 in the progression of CRC disease. In the current study, tumor suppressor lncRNA GAS5 was detected in exosomes derived from serum of CRC patients and non-cancer individuals. It was down-regulated (3-fold) in CRC patients as compared with non-cancer individuals. The results are in concordance with previous reports [40,41]. Functional studies revealed that lncRNA GAS5 acts as a tumor suppressor by inhibiting CRC cell proliferation, migration, and invasion [5,42]. In addition, low levels of GAS5 expression had a shorter survival time compared to those with high levels [5] and correlated with advanced TNM stages and larger tumor size [41]. Thus, our finding on the lower levels of lncRNA GAS5 in exosomes from sera of CRC patients is consistent with the tumor-suppressive function of lncRNA GAS5.

ZFAS1 has been reported to have an oncogenic role in various types of cancer, for example, bladder cancer [43], breast cancer [44,45], HCC [46], and CRC [47,48,49]. To date, there are no reports on the expression level of ZFAS1 in circulating exosomes derived from CRC. In the current study, the expression level in exosomes from sera of CRC patients is lower than non-cancer individuals (−5.12-fold, *p* = 0.09) and there is no difference in expression level between early and advanced-stage CRC (−1.45-fold, *p* = 0.69). In a study, the upregulation of ZFAS1 was reported to be correlated with lymphatic invasion and the promotion of invasion and metastasis in CRC [49]. This is the first report that revealed the expression level of ZFAS1 in exosomes derived from CRC patients. Future studies on the dynamics of ZFAS1 expression in the biogenesis of exosomes is worthy of further investigation.

In the year 2016, Christensen et al. demonstrated that the expression level of snoRNA host gene 16 (SNHG16) is high in all stages of CRC tissues and functional studies revealed that SNHG16 regulated cell viability and migration via the Wnt pathway in CRC [50]. Silencing of SNHG16 induced apoptosis and impaired cell migration consistent with its oncogenic role. Our results revealed that SNHG16 was down-regulated in exosomes from sera of CRC patients but there is no difference between early- and advanced-stage CRC. The reduced level of SNHG16 in exosomes from sera of CRC patients is in opposite trend to the reports with tissue samples. This opposite trend has also been shown by Barbagallo et al., who reported that lncRNA UCA1 was up-regulated in CRC tissues and down-regulated in serum exosomes [51]. However, it is unclear why the level of SNHG16 is lower in exosomes of sera from cancer patients versus non-cancer individuals. Further studies need to be carried out. LncRNA-RMRP has been shown to act as an oncogene in lung cancer [52]. To date, there are no clinical and *in vitro* studies on RMRP in CRC. The expression of this lncRNA was detected in all samples in the present study. However, there is no significant difference between CRC and non-cancer individuals. Similarly, there is no difference when comparing early- and advanced-stage CRC. Future studies need to be carried out with CRC tissues to determine the role of RMRP.

## 5. Conclusions

In conclusion, our study has shown that lncRNA, H19 was up-regulated in serum-derived exosomes from advanced-stage CRC, while exosomal LINC00152 was down-regulated in CRC as compared with non-cancer individuals. However, it should be noted that the expression level of exosomal H19 in 6 of the advanced-stage cases is low. LINC00152 was down-regulated in serum-derived exosomes from CRC patients, but, surprisingly 14 samples from non-cancer individuals also showed a low expression level similar to those from CRC patients. The mechanism underlying these differences is worthy of further investigation. This is the first report to show that H19 is differentially expressed in serum exosomes from early-stage versus advanced-stage CRC. Additionally, this is the first report on differentially expressed LINC00152 in exosomes from sera of CRC versus non-cancer patients. The roles of these exosomal lncRNAs in pathogenesis and progression of CRC needs further investigation.

## Figures and Tables

**Table 1:** PCR cycling condition.

Cycles	Duration	Temperature
1	10 min	95 °C
40	15 s	95 °C
	1 min	60 °C

**Table 2:** Clinical and pathological characteristics of colorectal cancer (CRC) patients included in the present study.

Clinico-Pathologic Parameters	Number of Patients (*n* = 18)
Sex	
Male	9
Female	9
Age (years)	
<60	8
≥60	10
Tumor stage	
I	1
II	7
III	7
IV	3
Tumor location	
Colon	6
Sigmoid	5
Rectosigmoid	2
Rectum	5

**Table 3:** The fold-change for lncRNAs GAS5, H19, LINC00152, RMRP, SNHG16, and ZFAS1 expression level in non-cancer individuals and CRC patients.

Target Genes	lncRNA Expression Level			
	Non-Cancer (*n* = 21)	CRC (*n* = 18)	*p*-Value	Fold-Change
GAS5	0.0708	0.2125	0.103	−3.00
H19	0.0255	0.0658	0.921	−2.58
LINC00152	0.0369	0.1190	0.040 *	−3.23
RMRP	0.0030	0.0070	0.246	−2.33
SNHG16	0.0015	0.0121	0.085	−8.06
ZFAS1	0.0230	0.0230	0.094	−5.12

*n,* number of samples. * indicates *p* < 0.05.

**Table 4:** The fold change for lncRNAs GAS5, H19, LINC00152, RMRP, SNHG16, and ZFAS1 expression level in exosomes of early-stages (stage I & II) and advanced stages (stage III & IV) of CRC patients.

Target Genes	lncRNAs Expression Level			
	Early Stages (*n* = 8)	Advanced Stages (*n* = 10)	*p*-Value	Fold Change
GAS5	0.0762	0.0646	0.942	1.18
H19	0.0957	0.0048	0.046 *	19.59
LINC00152	0.0256	0.0582	0.694	−2.27
RMRP	0.0025	0.0036	0.225	−1.44
SNHG16	0.0012	0.0019	0.899	1.62
ZFAS1	0.0171	0.0334	0.855	−1.95

*n,* number of samples. * indicates *p* < 0.05.

## Data Availability

Data is contained within the article or supplementary material.

## Supplementary Materials

The following are available online at https://www.mdpi.com/article/10.3390/biology10090918/s1, Table S1: Normalized gene expression level of lncRNAs for early versus advanced-stage of CRC patients. The *p* values are calculated based on a Student’s *t*-test of the replicate 2^(−ΔCt)^ values for each gene in the early-stage and advanced-stage of CRC patients, Table S2: Normalized gene expression level of lncRNAs for non-cancer individuals versus CRC patients. The *p* values are calculated based on a Student’s *t*-test of the replicate 2^(−ΔCt)^ values for each gene in the non-cancer group and CRC groups, Figure S1a: Western blot analysis of ALIX/PDCD6IP from exosomes derived from SW480-7 and total cell lysates of SW480-7. The chemiluminescencec image was captured at 1 min exposure time. Figure S1b: Western blot analysis of ALIX/PDCD6IP from exosomes derived from SW480-7 and total cell lysates of SW480-7. The chemiluminescence image was captured at 2 min exposure time. Figure S1c: Western blot analysis of ALIX/PDCD6IP from exosomes derived from SW480-7 and total cell lysates of SW480-7. The chemiluminescence image was captured at 3 min exposure time. Figure S1d: Western blot analysis of ALIX/PDCD6IP from exosomes derived from SW480-7 and total cell lysates of SW480-7. The chemiluminescence image was captured at 4 min exposure time. Figure S1e: Western blot analysis of ALIX/PDCD6IP from exosomes derived from SW480-7 and total cell lysates of SW480-7. The chemiluminescence image was captured at 5 min exposure time.

## References

[1] Moriarity A., O’Sullivan J., Kennedy J., Mehigan B., McCormick P. (2016). Current targeted therapies in the treatment of advanced colorectal cancer: A review. Ther. Adv. Med. Oncol..

[2] Arnold M., Sierra M.S., Laversanne M., Soerjomataram I., Jemal A., Bray F. (2017). Global patterns and trends in colorectal cancer incidence and mortality. Gut.

[3] Bray F., Ferlay J., Soerjomataram I., Siegel R.L., Torre L.A., Jemal A. (2018). Global cancer statistics 2018: GLOBOCAN estimates of incidence and mortality worldwide for 36 cancers in 185 countries. CA A Cancer J. Clin..

[4] Xie X., Tang B., Xiao Y.F., Xie R., Li B.S., Dong H., Zhou J.Y., Yang S.M. (2016). Long non-coding RNAs in colorectal cancer. Oncotarget.

[5] Saus E., Brunet-Vega A., Iraola-Guzman S., Pegueroles C., Gabaldon T., Pericay C. (2016). Long Non-Coding RNAs As Potential Novel Prognostic Biomarkers in Colorectal Cancer. Front. Genet..

[6] Han D., Wang M., Ma N., Xu Y., Jiang Y., Gao X. (2015). Long noncoding RNAs: Novel players in colorectal cancer. Cancer Lett..

[7] Ward M., McEwan C., Mills J.D., Janitz M. (2015). Conservation and tissue-specific transcription patterns of long noncoding RNAs. J. Hum. Transcr..

[8] Raut S.K., Khullar M. (2018). The Big Entity of New RNA World: Long Non-Coding RNAs in Microvascular Complications of Diabetes. Front. Endocrinol..

[9] Balas M.M., Johnson A.M. (2018). Exploring the mechanisms behind long noncoding RNAs and cancer. Non-Coding Rna Res..

[10] Smolle M., Uranitsch S., Gerger A., Pichler M., Haybaeck J. (2014). Current status of long non-coding RNAs in human cancer with specific focus on colorectal cancer. Int. J. Mol. Sci..

[11] Ye L.C., Chen T., Zhu D.X., Lv S.X., Qiu J.J., Xu J., Yuan F.L., Wei Y. (2016). Downregulated long non-coding RNA CLMAT3 promotes the proliferation of colorectal cancer cells by targeting regulators of the cell cycle pathway. Oncotarget.

[12] Yin D.D., Liu Z.J., Zhang E., Kong R., Zhang Z.H., Guo R.H. (2015). Decreased expression of long noncoding RNA MEG3 affects cell proliferation and predicts a poor prognosis in patients with colorectal cancer. Tumor Biol. J. Int. Soc. Oncodev. Biol. Med..

[13] Takahashi Y., Sawada G., Kurashige J., Uchi R., Matsumura T., Ueo H., Takano Y., Eguchi H., Sudo T., Sugimachi K. (2014). Amplification of PVT-1 is involved in poor prognosis via apoptosis inhibition in colorectal cancers. Br. J. Cancer.

[14] Qi P., Xu M.D., Ni S.J., Shen X.H., Wei P., Huang D., Tan C., Sheng W.Q., Zhou X.Y., Du X. (2015). Down-regulation of ncRAN, a long non-coding RNA, contributes to colorectal cancer cell migration and invasion and predicts poor overall survival for colorectal cancer patients. Mol. Carcinog..

[15] Roma-Rodrigues C., Fernandes A.R., Baptista P.V. (2014). Exosome in tumor microenvironment: Overview of the crosstalk between normal and cancer cells. Biomed. Res. Int..

[16] Li J., Xue W., Lv J., Han P., Liu Y., Cui B. (2017). Identification of potential long non-coding RNA biomarkers associated with the progression of colon cancer. Oncotarget.

[17] Yokoi A., Yoshioka Y., Hirakawa A., Yamamoto Y., Ishikawa M., Ikeda S.I., Kato T., Niimi K., Kajiyama H., Kikkawa F. (2017). A combination of circulating miRNAs for the early detection of ovarian cancer. Oncotarget.

[18] Farran B., Dyson G., Craig D., Dombkowski A., Beebe-Dimmer J.L., Powell I.J., Podgorski I., Heilbrun L., Bolton S., Bock C.H. (2018). A study of circulating microRNAs identifies a new potential biomarker panel to distinguish aggressive prostate cancer. Carcinogenesis.

[19] Fang Z., Tang J., Bai Y., Lin H., You H., Jin H., Lin L., You P., Li J., Dai Z. (2015). Plasma levels of microRNA-24, microRNA-320a, and microRNA-423-5p are potential biomarkers for colorectal carcinoma. J. Exp. Clin. Cancer Res. Cr.

[20] Zhang J., Raju G.S., Chang D.W., Lin S.H., Chen Z., Wu X. (2018). Global and targeted circulating microRNA profiling of colorectal adenoma and colorectal cancer. Cancer.

[21] Zheng G., Du L., Yang X., Zhang X., Wang L., Yang Y., Li J., Wang C. (2014). Serum microRNA panel as biomarkers for early diagnosis of colorectal adenocarcinoma. Br. J. Cancer.

[22] Sapre N., Selth L.A. (2013). Circulating MicroRNAs as Biomarkers of Prostate Cancer: The State of Play. Prostate Cancer.

[23] Hamam R., Hamam D., Alsaleh K.A., Kassem M., Zaher W., Alfayez M., Aldahmash A., Alajez N.M. (2017). Circulating microRNAs in breast cancer: Novel diagnostic and prognostic biomarkers. Cell Death Dis..

[24] Dragomir M., Chen B., Calin G.A. (2018). Exosomal lncRNAs as new players in cell-to-cell communication. Transl. Cancer Res..

[25] Wu Y., Wang Y., Wei M., Han X., Xu T., Cui M. (2020). Advances in the study of exosomal lncRNAs in tumors and the selection of research methods. Biomed. Pharmacother..

[26] He P.Y., Yip W.K., Chai B.L., Chai B.Y., Jabar M.F., Dusa N., Mohtarrudin N., Seow H.F. (2017). Inhibition of cell migration and invasion by miR-29a-3p in a colorectal cancer cell line through suppression of CDC42BPA mRNA expression. Oncol. Rep..

[27] Enderle D., Spiel A., Coticchia C.M., Berghoff E., Mueller R., Schlumpberger M., Sprenger-Haussels M., Shaffer J.M., Lader E., Skog J. (2015). Characterization of RNA from Exosomes and Other Extracellular Vesicles Isolated by a Novel Spin Column-Based Method. PLoS ONE.

[28] Tickner J.A., Urquhart A.J., Stephenson S.A., Richard D.J., O’Byrne K.J. (2014). Functions and therapeutic roles of exosomes in cancer. Front. Oncol..

[29] Wortzel I., Dror S., Kenific C.M., Lyden D. (2019). Exosome-Mediated Metastasis: Communication from a Distance. Dev. Cell.

[30] Feng W., Dean D.C., Hornicek F.J., Shi H., Duan Z. (2019). Exosomes promote pre-metastatic niche formation in ovarian cancer. Mol. Cancer.

[31] Guo Y., Ji X., Liu J., Fan D., Zhou Q., Chen C., Wang W., Wang G., Wang H., Yuan W. (2019). Effects of exosomes on pre-metastatic niche formation in tumors. Mol. Cancer.

[32] Liu T., Zhang X., Gao S., Jing F., Yang Y., Du L., Zheng G., Li P., Li C., Wang C. (2016). Exosomal long noncoding RNA CRNDE-h as a novel serum-based biomarker for diagnosis and prognosis of colorectal cancer. Oncotarget.

[33] Tang Y.T., Huang Y.Y., Zheng L., Qin S.H., Xu X.P., An T.X., Xu Y., Wu Y.S., Hu X.M., Ping B.H. (2017). Comparison of isolation methods of exosomes and exosomal RNA from cell culture medium and serum. Int. J. Mol. Med..

[34] Yu Y., Yang J., Li Q., Xu B., Lian Y., Miao L. (2017). LINC00152: A pivotal oncogenic long non-coding RNA in human cancers. Cell Prolif..

[35] Ou C., Sun Z., He X., Li X., Fan S., Zheng X., Peng Q., Li G., Li X., Ma J. (2020). Targeting YAP1/LINC00152/FSCN1 Signaling Axis Prevents the Progression of Colorectal Cancer. Adv. Sci. (Weinh. Baden-Wurtt. Ger.).

[36] Zhang Y.H., Fu J., Zhang Z.J., Ge C.C., Yi Y. (2016). LncRNA-LINC00152 down-regulated by miR-376c-3p restricts viability and promotes apoptosis of colorectal cancer cells. Am. J. Transl. Res..

[37] Delihas N. (2013). Editorial on the Special Issue: Regulation by non-coding RNAs. Int. J. Mol. Sci..

[38] Li Q., Dai Y., Wang F., Hou S. (2016). Differentially expressed long non-coding RNAs and the prognostic potential in colorectal cancer. Neoplasma.

[39] Deng Q., He B., Gao T., Pan Y., Sun H., Xu Y., Li R., Ying H., Wang F., Liu X. (2014). Up-regulation of 91H promotes tumor metastasis and predicts poor prognosis for patients with colorectal cancer. PLoS ONE.

[40] Liu L., Meng T., Yang X.H., Sayim P., Lei C., Jin B., Ge L., Wang H.J. (2018). Prognostic and predictive value of long non-coding RNA GAS5 and mircoRNA-221 in colorectal cancer and their effects on colorectal cancer cell proliferation, migration and invasion. Cancer Biomark. Sect. A Dis. Markers.

[41] Galamb O., Barták B.K., Kalmár A., Nagy Z.B., Szigeti K.A., Tulassay Z., Igaz P., Molnár B. (2019). Diagnostic and prognostic potential of tissue and circulating long non-coding RNAs in colorectal tumors. World J. Gastroenterol..

[42] Xie Y., Dang W., Zhang S., Yue W., Yang L., Zhai X., Yan Q., Lu J. (2019). The role of exosomal noncoding RNAs in cancer. Mol. Cancer.

[43] Wang J.S., Liu Q.H., Cheng X.H., Zhang W.Y., Jin Y.C. (2018). The long noncoding RNA ZFAS1 facilitates bladder cancer tumorigenesis by sponging miR-329. Biomed. Pharmacother..

[44] Fan S., Fan C., Liu N., Huang K., Fang X., Wang K. (2018). Downregulation of the long non-coding RNA ZFAS1 is associated with cell proliferation, migration and invasion in breast cancer. Mol. Med. Rep..

[45] Hajjari M., Salavaty A. (2015). HOTAIR: An oncogenic long non-coding RNA in different cancers. Cancer Biol. Med..

[46] Luo P., Liang C., Zhang X., Liu X., Wang Y., Wu M., Feng X., Tu J. (2018). Identification of long non-coding RNA ZFAS1 as a novel biomarker for diagnosis of HCC. Biosci. Rep..

[47] Chen X., Zeng K., Xu M., Hu X., Liu X., Xu T., He B., Pan Y., Sun H., Wang S. (2018). SP1-induced lncRNA-ZFAS1 contributes to colorectal cancer progression via the miR-150-5p/VEGFA axis. Cell Death Dis..

[48] Thorenoor N., Faltejskova-Vychytilova P., Hombach S., Mlcochova J., Kretz M., Svoboda M., Slaby O. (2016). Long non-coding RNA ZFAS1 interacts with CDK1 and is involved in p53-dependent cell cycle control and apoptosis in colorectal cancer. Oncotarget.

[49] Wang W., Xing C. (2016). Upregulation of long noncoding RNA ZFAS1 predicts poor prognosis and prompts invasion and metastasis in colorectal cancer. Pathol. Res. Pract..

[50] Christensen L.L., True K., Hamilton M.P., Nielsen M.M., Damas N.D., Damgaard C.K., Ongen H., Dermitzakis E., Bramsen J.B., Pedersen J.S. (2016). SNHG16 is regulated by the Wnt pathway in colorectal cancer and affects genes involved in lipid metabolism. Mol. Oncol..

[51] Barbagallo C., Brex D., Caponnetto A., Cirnigliaro M., Scalia M., Magnano A., Caltabiano R., Barbagallo D., Biondi A., Cappellani A. (2018). LncRNA UCA1, Upregulated in CRC Biopsies and Downregulated in Serum Exosomes, Controls mRNA Expression by RNA-RNA Interactions. Mol. Ther.-Nucleic Acids.

[52] Meng Q., Ren M., Li Y., Song X. (2016). LncRNA-RMRP Acts as an Oncogene in Lung Cancer. PLoS ONE.

