# Supplementary material for: Isolation and Identification of Long Non-Coding RNAs in Exosomes Derived from the Serum of Colorectal Carcinoma Patients
Source: Biology (Basel). 2021 Sep 15;10(9):918. doi: 10.3390/biology10090918 (PMC8465981; doi:10.3390/biology10090918)
Supplement: Supplementary file 1 [file biology-10-00918-s001.zip › biology-878372-supplementary.pdf]

**Table S1.** Normalized gene expression level of lncRNAs for early versus advanced-stage of CRC patients. The *p* values are calculated based on a Student's *t*-test of the replicate 2^ (-Delta Ct) values for each gene in the early-stage and advanced-stage of CRC patients.

| Position | Gene Symbol | AVG $\Delta C_t$ | | 2^-$\Delta C_t$ | | Fold Change | | *p*-value | Fold Up- or Down-Regulation |
|---|---|---|---|---|---|---|---|---|---|
| | | Advanced-stage | Early-stage | Advanced-stage | Early-stage | Early-stage/advanced-stage | Comments | Early-stage/advanced-stage | Early stage/advanced-stage |
| A01 | ACTA2-AS1 | 12.13 | 11.71 | 0.000223 | 0.000297 | 0.75 | C | 0.350699 | −1.33 |
| A02 | ADAMTS9-AS2 | 12.13 | 11.71 | 0.000223 | 0.000298 | 0.75 | B | 0.350293 | −1.34 |
| A03 | AFAP1-AS1 | 12.06 | 11.71 | 0.000233 | 0.000297 | 0.78 | B | 0.352468 | −1.27 |
| A04 | AIRN | 12.13 | 11.71 | 0.000223 | 0.000297 | 0.75 | C | 0.350699 | −1.33 |
| A05 | BANCR | 12.13 | 11.71 | 0.000223 | 0.000297 | 0.75 | C | 0.350699 | −1.33 |
| A06 | BCAR4 | 12.13 | 11.71 | 0.000223 | 0.000297 | 0.75 | C | 0.350699 | −1.33 |
| A07 | BLACAT1 | 12.13 | 11.71 | 0.000223 | 0.000297 | 0.75 | C | 0.350699 | −1.33 |
| A08 | CAHM | 12.13 | 11.71 | 0.000223 | 0.000297 | 0.75 | C | 0.350699 | −1.33 |
| A09 | CBR3-AS1 | 12.13 | 11.71 | 0.000223 | 0.000297 | 0.75 | C | 0.350699 | −1.33 |
| A10 | CCAT1 | 12.13 | 11.71 | 0.000223 | 0.000297 | 0.75 | C | 0.350699 | −1.33 |
| A11 | CCAT2 | 12.11 | 11.71 | 0.000227 | 0.000297 | 0.76 | B | 0.352671 | −1.31 |
| A12 | CDKN2B−AS1 | 12.13 | 11.08 | 0.000223 | 0.000462 | 0.48 | B | 0.226005 | −2.07 |
| B01 | CRNDE | 11.64 | 11.44 | 0.000314 | 0.000361 | 0.87 | B | 0.590460 | −1.15 |
| B02 | DGCR5 | 12.13 | 11.48 | 0.000223 | 0.000349 | 0.64 | B | 0.314541 | −1.56 |
| B03 | DLEU2 | 11.59 | 10.5 | 0.000325 | 0.00069 | 0.47 | B | 0.064086 | −2.12 |
| B04 | DLX6-AS1 | 12.13 | 11.71 | 0.000223 | 0.000297 | 0.75 | C | 0.350699 | −1.33 |
| B05 | EMX2OS | 12.13 | 11.71 | 0.000223 | 0.000297 | 0.75 | C | 0.350699 | −1.33 |
| B06 | FTX | 12.08 | 11.71 | 0.000231 | 0.000297 | 0.78 | B | 0.369633 | −1.29 |
| B07 | GACAT1 | 12.13 | 11.71 | 0.000223 | 0.000297 | 0.75 | C | 0.350699 | −1.33 |
| B08 | GAS5 | 3.71 | 3.95 | 0.076203 | 0.064648 | 1.18 | | 0.942845 | 1.18 |
| B09 | GAS6-AS1 | 12.13 | 11.71 | 0.000223 | 0.000297 | 0.75 | C | 0.350699 | −1.33 |
| B10 | GNAS-AS1 | 12.13 | 11.71 | 0.000223 | 0.000297 | 0.75 | C | 0.350699 | −1.33 |
| B11 | H19 | 3.38 | 7.68 | 0.095789 | 0.004889 | 19.59 | | 0.046517 | 19.59 |
| B12 | HAND2-AS1 | 12.13 | 11.61 | 0.000223 | 0.00032 | 0.7 | B | 0.335190 | −1.43 |
| C01 | HEIH | 10.1 | 9.73 | 0.000912 | 0.001174 | 0.78 | B | 0.572422 | −1.29 |
| C02 | HIF1A-AS1 | 12.13 | 11.71 | 0.000223 | 0.000297 | 0.75 | C | 0.350699 | −1.33 |
| C03 | HIF1A-AS2 | 12.13 | 11.71 | 0.000223 | 0.000297 | 0.75 | C | 0.350699 | −1.33 |
| C04 | HNF1A-AS1 | 12.13 | 11.52 | 0.000223 | 0.000341 | 0.65 | B | 0.322718 | −1.53 |
| C05 | HOTAIR | 12.13 | 11.51 | 0.000223 | 0.000343 | 0.65 | B | 0.321589 | −1.53 |
| C06 | HOTAIRM1 | 11.85 | 9.37 | 0.000272 | 0.001509 | 0.18 | B | 0.121880 | −5.55 |
| C07 | HOTTIP | 12.13 | 11.71 | 0.000223 | 0.000297 | 0.75 | C | 0.350699 | −1.33 |
| C08 | HOXA11-AS | 12.13 | 11.71 | 0.000223 | 0.000297 | 0.75 | C | 0.350699 | −1.33 |
| C09 | HOXA-AS2 | 12.13 | 11.71 | 0.000223 | 0.000297 | 0.75 | C | 0.350699 | −1.33 |
| C10 | HULC | 12.13 | 11.71 | 0.000223 | 0.000297 | 0.75 | C | 0.350699 | −1.33 |

| | | | | | | | | | |
|---|---|---|---|---|---|---|---|---|---|
| C11 | IPW | 12.13 | 11.22 | 0.000223 | 0.000419 | 0.53 | B | 0.163563 | −1.87 |
| C12 | JADRR | 12.13 | 11.61 | 0.000223 | 0.000321 | 0.7 | B | 0.338916 | −1.44 |
| D01 | KCNQ1OT1 | 12.13 | 11.18 | 0.000223 | 0.000432 | 0.52 | B | 0.173818 | −1.93 |
| D02 | KRASP1 | 12.13 | 11.71 | 0.000223 | 0.000297 | 0.75 | C | 0.350699 | −1.33 |
| D03 | LINC00152 | 5.29 | 4.1 | 0.02563 | 0.058264 | 0.44 | | 0.694516 | −2.27 |
| D04 | LINC00261 | 12.13 | 11.43 | 0.000223 | 0.000362 | 0.62 | B | 0.299397 | −1.62 |
| D05 | LINC00312 | 12.13 | 11.43 | 0.000223 | 0.000362 | 0.62 | B | 0.299397 | −1.62 |
| D06 | LINC00538 | 12.13 | 11.71 | 0.000223 | 0.000297 | 0.75 | C | 0.350699 | −1.33 |
| D07 | LINC00887 | 12.13 | 11.71 | 0.000223 | 0.000297 | 0.75 | C | 0.350699 | −1.33 |
| D08 | LINC00963 | 12.13 | 11.54 | 0.000223 | 0.000336 | 0.67 | B | 0.320303 | −1.5 |
| D09 | LINC01233 | 12.13 | 11.71 | 0.000223 | 0.000297 | 0.75 | C | 0.350699 | −1.33 |
| D10 | LINC01234 | 12.13 | 11.71 | 0.000223 | 0.000297 | 0.75 | C | 0.350699 | −1.33 |
| D11 | LSINCT5 | 12.13 | 11.65 | 0.000223 | 0.000311 | 0.72 | B | 0.344617 | −1.39 |
| D12 | LUCAT1 | 12.08 | 11.71 | 0.000231 | 0.000297 | 0.78 | B | 0.360726 | −1.29 |
| E01 | MALAT1 | 12.13 | 11.71 | 0.000223 | 0.000297 | 0.75 | C | 0.350699 | −1.33 |
| E02 | MEG3 | 12.13 | 11.52 | 0.000223 | 0.000342 | 0.65 | B | 0.322439 | −1.53 |
| E03 | MIR155HG | 11.9 | 11.7 | 0.000262 | 0.000301 | 0.87 | B | 0.660549 | −1.15 |
| E04 | MIR17HG | 12.13 | 11.71 | 0.000223 | 0.000297 | 0.75 | C | 0.350699 | −1.33 |
| E05 | MIR31HG | 12.13 | 11.71 | 0.000223 | 0.000298 | 0.75 | B | 0.350498 | −1.33 |
| E06 | MIR7-3HG | 12.13 | 11.71 | 0.000223 | 0.000297 | 0.75 | C | 0.350699 | −1.33 |
| E07 | MRPL23-AS1 | 12.13 | 11.71 | 0.000223 | 0.000297 | 0.75 | C | 0.350699 | −1.33 |
| E08 | NAMA | 12.13 | 11.62 | 0.000223 | 0.000317 | 0.7 | B | 0.340966 | −1.42 |
| E09 | NBR2 | 11.85 | 11.57 | 0.000272 | 0.000328 | 0.83 | B | 0.671969 | −1.21 |
| E10 | NEAT1 | 9.78 | 8.86 | 0.001141 | 0.002147 | 0.53 | B | 0.842517 | −1.88 |
| E11 | NRON | 12.13 | 11.71 | 0.000223 | 0.000297 | 0.75 | C | 0.350699 | −1.33 |
| E12 | PANDAR | 12.13 | 11.71 | 0.000223 | 0.000297 | 0.75 | C | 0.350699 | −1.33 |
| F01 | PCA3 | 12.13 | 11.71 | 0.000223 | 0.000297 | 0.75 | C | 0.350699 | −1.33 |
| F02 | PCAT1 | 12.13 | 11.71 | 0.000223 | 0.000297 | 0.75 | C | 0.350699 | −1.33 |
| F03 | PCGEM1 | 12.13 | 11.71 | 0.000223 | 0.000297 | 0.75 | C | 0.350699 | −1.33 |
| F04 | POU5F1P5 | 12.13 | 11.71 | 0.000223 | 0.000297 | 0.75 | C | 0.350699 | −1.33 |
| F05 | PRNCR1 | 11.84 | 11.27 | 0.000273 | 0.000406 | 0.67 | B | 0.290353 | −1.49 |
| F06 | PTCSC1 | 12.13 | 11.71 | 0.000223 | 0.000297 | 0.75 | C | 0.350699 | −1.33 |
| F07 | PTCSC3 | 12.13 | 11.57 | 0.000223 | 0.00033 | 0.68 | B | 0.332392 | −1.48 |
| F08 | PTENP1 | 11.99 | 11.71 | 0.000245 | 0.000297 | 0.82 | B | 0.393893 | −1.21 |
| F09 | PVT1 | 11.9 | 10.77 | 0.000261 | 0.000575 | 0.45 | B | 0.182207 | −2.2 |
| F10 | RMRP | 8.6 | 8.08 | 0.002572 | 0.003699 | 0.7 | | 0.225709 | −1.44 |
| F11 | RMST | 12.13 | 11.71 | 0.000223 | 0.000297 | 0.75 | C | 0.350699 | −1.33 |
| F12 | RPS6KA2-AS1 | 12.13 | 11.71 | 0.000223 | 0.000297 | 0.75 | C | 0.350699 | −1.33 |
| G01 | SNHG16 | 9.68 | 8.98 | 0.001218 | 0.001977 | 0.62 | B | 0.899145 | −1.62 |
| G02 | SPRY4-IT1 | 12.13 | 11.71 | 0.000223 | 0.000297 | 0.75 | C | 0.350699 | −1.33 |
| G03 | SUMO1P3 | 12.13 | 11.71 | 0.000223 | 0.000297 | 0.75 | C | 0.350699 | −1.33 |
| G04 | TERC | 12.13 | 11.6 | 0.000223 | 0.000322 | 0.69 | B | 0.338378 | −1.44 |
| G05 | TRERNA1 | 12.13 | 11.57 | 0.000223 | 0.000329 | 0.68 | B | 0.303832 | −1.47 |

| | | | | | | | | | |
|---|---|---|---|---|---|---|---|---|---|
| G06 | TSIX | 12.13 | 11.71 | 0.000223 | 0.000297 | 0.75 | C | 0.350699 | −1.33 |
| G07 | TUG1 | 11.67 | 10.09 | 0.000308 | 0.00092 | 0.33 | | 0.025613 | −2.99 |
| G08 | TUSC7 | 12.13 | 11.71 | 0.000223 | 0.000297 | 0.75 | C | 0.350699 | −1.33 |
| G09 | UCA1 | 12.13 | 11.13 | 0.000223 | 0.000447 | 0.5 | B | 0.131656 | −2 |
| G10 | WT1−AS | 12.13 | 11.59 | 0.000223 | 0.000325 | 0.69 | B | 0.335924 | −1.46 |
| G11 | XIST | 12.11 | 11.7 | 0.000227 | 0.000301 | 0.75 | B | 0.356766 | −1.33 |
| G12 | ZFAS1 | 5.87 | 4.9 | 0.017122 | 0.033464 | 0.51 | | 0.855877 | −1.95 |

**Table S2.** Normalized gene expression level of lncRNAs for non-cancer individuals versus CRC patients. The p values are calculated based on a Student's *t*-test of the replicate 2^ (- Delta Ct) values for each gene in the non-cancer group and CRC groups.

| Position | Gene Symbol | AVG $\Delta C_t$ | | $2^{-\Delta C_t}$ | | Fold Change | | *p*-value | Fold Up-or Down-Regulation |
|---|---|---|---|---|---|---|---|---|---|
| | | CRC | Non-cancer | CRC | Non-cancer | CRC /non-cancer | Comments | CRC /non-cancer | CRC /non-cancer |
| A01 | ACTA2-AS1 | 11.94 | 10.82 | 0.000254 | 0.000552 | 0.46 | C | 0.054175 | −2.18 |
| A02 | ADAMTS9-AS2 | 11.94 | 10.81 | 0.000254 | 0.000556 | 0.46 | B | 0.052259 | −2.19 |
| A03 | AFAP1-AS1 | 11.91 | 10.75 | 0.00026 | 0.000579 | 0.45 | B | 0.053479 | −2.23 |
| A04 | AIRN | 11.94 | 10.76 | 0.000254 | 0.000578 | 0.44 | B | 0.057746 | −2.28 |
| A05 | BANCR | 11.94 | 10.82 | 0.000254 | 0.000552 | 0.46 | C | 0.054175 | −2.18 |
| A06 | BCAR4 | 11.94 | 10.71 | 0.000254 | 0.000597 | 0.42 | B | 0.061246 | −2.35 |
| A07 | BLACAT1 | 11.94 | 10.77 | 0.000254 | 0.000574 | 0.44 | | 0.041756 | −2.26 |
| A08 | CAHM | 11.94 | 10.7 | 0.000254 | 0.000603 | 0.42 | | 0.034852 | −2.38 |
| A09 | CBR3-AS1 | 11.94 | 10.82 | 0.000254 | 0.000552 | 0.46 | C | 0.054175 | −2.18 |
| A10 | CCAT1 | 11.94 | 10.82 | 0.000254 | 0.000552 | 0.46 | C | 0.054175 | −2.18 |
| A11 | CCAT2 | 11.93 | 10.82 | 0.000256 | 0.000554 | 0.46 | B | 0.054127 | −2.17 |
| A12 | CDKN2B-AS1 | 11.66 | 10.82 | 0.000309 | 0.000552 | 0.56 | B | 0.667772 | −1.79 |
| B01 | CRNDE | 11.55 | 10.67 | 0.000334 | 0.000616 | 0.54 | B | 0.889903 | −1.84 |
| B02 | DGCR5 | 11.84 | 10.82 | 0.000273 | 0.000552 | 0.49 | B | 0.057720 | −2.03 |
| B03 | DLEU2 | 11.1 | 9.66 | 0.000454 | 0.001239 | 0.37 | B | 0.118556 | −2.73 |
| B04 | DLX6-AS1 | 11.94 | 10.82 | 0.000254 | 0.000552 | 0.46 | C | 0.054175 | −2.18 |
| B05 | EMX2OS | 11.94 | 10.8 | 0.000254 | 0.000561 | 0.45 | B | 0.053222 | −2.21 |
| B06 | FTX | 11.92 | 10.38 | 0.000258 | 0.000751 | 0.34 | B | 0.200192 | −2.91 |
| B07 | GACAT1 | 11.94 | 10.8 | 0.000254 | 0.000559 | 0.45 | B | 0.053289 | −2.2 |
| B08 | GAS5 | 3.82 | 2.23 | 0.070833 | 0.212526 | 0.33 | | 0.103149 | −3 |
| B09 | GAS6-AS1 | 11.94 | 10.81 | 0.000254 | 0.000556 | 0.46 | B | 0.051232 | −2.19 |
| B10 | GNAS-AS1 | 11.94 | 10.82 | 0.000254 | 0.000552 | 0.46 | C | 0.054175 | −2.18 |
| B11 | H19 | 5.29 | 3.92 | 0.02553 | 0.065889 | 0.39 | | 0.921921 | −2.58 |
| B12 | HAND2-AS1 | 11.9 | 10.82 | 0.000262 | 0.000552 | 0.47 | B | 0.055638 | −2.11 |
| C01 | HEIH | 9.94 | 7.97 | 0.001021 | 0.003978 | 0.26 | B | 0.772519 | −3.9 |
| C02 | HIF1A-AS1 | 11.94 | 9.43 | 0.000254 | 0.001452 | 0.17 | B | 0.361454 | −5.72 |
| C03 | HIF1A-AS2 | 11.94 | 10.82 | 0.000254 | 0.000552 | 0.46 | C | 0.054175 | −2.18 |
| C04 | HNF1A-AS1 | 11.86 | 10.76 | 0.00027 | 0.000576 | 0.47 | B | 0.053025 | −2.14 |
| C05 | HOTAIR | 11.85 | 10.71 | 0.00027 | 0.000598 | 0.45 | | 0.044996 | −2.21 |

| | | | | | | | | | |
|---|---|---|---|---|---|---|---|---|---|
| C06 | HOTAIRM1 | 10.75 | 10.11 | 0.000582 | 0.000905 | 0.64 | B | 0.730295 | −1.56 |
| C07 | HOTTIP | 11.94 | 10.73 | 0.000254 | 0.000588 | 0.43 | B | 0.055283 | −2.32 |
| C08 | HOXA11-AS | 11.94 | 10.82 | 0.000254 | 0.000552 | 0.46 | C | 0.054175 | −2.18 |
| C09 | HOXA-AS2 | 11.94 | 10.82 | 0.000254 | 0.000552 | 0.46 | C | 0.054175 | −2.18 |
| C10 | HULC | 11.94 | 10.78 | 0.000254 | 0.000567 | 0.45 | B | 0.050711 | −2.24 |
| C11 | IPW | 11.73 | 9.82 | 0.000295 | 0.001107 | 0.27 | B | 0.109129 | −3.75 |
| C12 | JADRR | 11.9 | 10.76 | 0.000262 | 0.000575 | 0.46 | | 0.047305 | −2.19 |
| D01 | KCNQ1OT1 | 11.71 | 10.82 | 0.000299 | 0.000552 | 0.54 | B | 0.090097 | −1.85 |
| D02 | KRASP1 | 11.94 | 10.82 | 0.000254 | 0.000552 | 0.46 | C | 0.054175 | −2.18 |
| D03 | LINC00152 | 4.76 | 3.07 | 0.03692 | 0.11908 | 0.31 | | 0.040188 | −3.23 |
| D04 | LINC00261 | 11.82 | 10.74 | 0.000277 | 0.000584 | 0.47 | | 0.048652 | −2.11 |
| D05 | LINC00312 | 11.82 | 10.81 | 0.000277 | 0.000559 | 0.5 | B | 0.058608 | −2.02 |
| D06 | LINC00538 | 11.94 | 10.71 | 0.000254 | 0.000596 | 0.43 | B | 0.054117 | −2.35 |
| D07 | LINC00887 | 11.94 | 10.8 | 0.000254 | 0.00056 | 0.45 | B | 0.053868 | −2.21 |
| D08 | LINC00963 | 11.87 | 9.97 | 0.000268 | 0.001 | 0.27 | B | 0.172212 | −3.73 |
| D09 | LINC01233 | 11.94 | 10.82 | 0.000254 | 0.000552 | 0.46 | C | 0.054175 | −2.18 |
| D10 | LINC01234 | 11.94 | 10.82 | 0.000254 | 0.000552 | 0.46 | C | 0.054175 | −2.18 |
| D11 | LSINCT5 | 11.92 | 10.81 | 0.000259 | 0.000556 | 0.47 | B | 0.054068 | −2.15 |
| D12 | LUCAT1 | 11.92 | 10.78 | 0.000259 | 0.00057 | 0.45 | B | 0.051017 | −2.2 |
| E01 | MALAT1 | 11.94 | 10.8 | 0.000254 | 0.000563 | 0.45 | B | 0.055227 | −2.22 |
| E02 | MEG3 | 11.86 | 10.82 | 0.00027 | 0.000552 | 0.49 | B | 0.056877 | −2.05 |
| E03 | MIR155HG | 11.81 | 10.7 | 0.000279 | 0.000601 | 0.46 | B | 0.101757 | −2.16 |
| E04 | MIR17HG | 11.94 | 10.82 | 0.000254 | 0.000552 | 0.46 | C | 0.054175 | −2.18 |
| E05 | MIR31HG | 11.94 | 10.77 | 0.000254 | 0.000572 | 0.44 | B | 0.053014 | −2.25 |
| E06 | MIR7-3HG | 11.94 | 9.66 | 0.000254 | 0.001239 | 0.2 | B | 0.361451 | −4.88 |
| E07 | MRPL23-AS1 | 11.94 | 10.82 | 0.000254 | 0.000552 | 0.46 | C | 0.054175 | −2.18 |
| E08 | NAMA | 11.9 | 10.81 | 0.000261 | 0.000556 | 0.47 | B | 0.052384 | −2.13 |
| E09 | NBR2 | 11.72 | 9.92 | 0.000296 | 0.001035 | 0.29 | B | 0.075069 | −3.5 |
| E10 | NEAT1 | 9.37 | 7.87 | 0.001511 | 0.004273 | 0.35 | B | 0.252364 | −2.83 |
| E11 | NRON | 11.94 | 10.82 | 0.000254 | 0.000552 | 0.46 | C | 0.054175 | −2.18 |
| E12 | PANDAR | 11.94 | 10.82 | 0.000254 | 0.000553 | 0.46 | B | 0.053701 | −2.18 |
| F01 | PCA3 | 11.94 | 10.81 | 0.000254 | 0.000557 | 0.46 | B | 0.050573 | −2.2 |
| F02 | PCAT1 | 11.94 | 10.82 | 0.000254 | 0.000552 | 0.46 | C | 0.054175 | −2.18 |
| F03 | PCGEM1 | 11.94 | 10.82 | 0.000254 | 0.000552 | 0.46 | C | 0.054175 | −2.18 |
| F04 | POU5F1P5 | 11.94 | 10.65 | 0.000254 | 0.000623 | 0.41 | B | 0.051159 | −2.46 |
| F05 | PRNCR1 | 11.58 | 9.86 | 0.000326 | 0.001074 | 0.3 | B | 0.072399 | −3.3 |
| F06 | PTCSC1 | 11.94 | 10.8 | 0.000254 | 0.000559 | 0.45 | B | 0.052811 | −2.2 |
| F07 | PTCSC3 | 11.88 | 10.75 | 0.000266 | 0.000582 | 0.46 | B | 0.079524 | −2.19 |
| F08 | PTENP1 | 11.87 | 10.8 | 0.000267 | 0.000559 | 0.48 | B | 0.058380 | −2.09 |
| F09 | PVT1 | 11.4 | 9.42 | 0.000371 | 0.001459 | 0.25 | B | 0.325096 | −3.94 |
| F10 | RMRP | 8.37 | 7.15 | 0.003023 | 0.007046 | 0.43 | | 0.246914 | −2.33 |
| F11 | RMST | 11.94 | 10.7 | 0.000254 | 0.000599 | 0.42 | | 0.037760 | −2.36 |
| F12 | RPS6KA2-AS1 | 11.94 | 10.82 | 0.000254 | 0.000552 | 0.46 | C | 0.054175 | −2.18 |

| | | | | | | | | | |
|---|---|---|---|---|---|---|---|---|---|
| G01 | SNHG16 | 9.37 | 6.36 | 0.001511 | 0.012182 | 0.12 | A | 0.085807 | −8.06 |
| G02 | SPRY4-IT1 | 11.94 | 10.82 | 0.000254 | 0.000552 | 0.46 | C | 0.054175 | −2.18 |
| G03 | SUMO1P3 | 11.94 | 10.78 | 0.000254 | 0.000568 | 0.45 | | 0.046959 | −2.24 |
| G04 | TERC | 11.89 | 10.78 | 0.000263 | 0.000568 | 0.46 | | 0.047714 | −2.16 |
| G05 | TRERNA1 | 11.88 | 10.8 | 0.000265 | 0.00056 | 0.47 | B | 0.058883 | −2.11 |
| G06 | TSIX | 11.94 | 10.81 | 0.000254 | 0.000555 | 0.46 | B | 0.053604 | −2.19 |
| G07 | TUG1 | 10.96 | 9.5 | 0.0005 | 0.001379 | 0.36 | B | 0.184977 | −2.76 |
| G08 | TUSC7 | 11.94 | 10.82 | 0.000254 | 0.000552 | 0.46 | C | 0.054175 | −2.18 |
| G09 | UCA1 | 11.68 | 10.64 | 0.000304 | 0.000628 | 0.48 | B | 0.075796 | −2.06 |
| G10 | WT1-AS | 11.89 | 10.82 | 0.000264 | 0.000555 | 0.48 | B | 0.053505 | −2.1 |
| G11 | XIST | 11.93 | 10.77 | 0.000257 | 0.000573 | 0.45 | B | 0.050035 | −2.23 |
| G12 | ZFAS1 | 5.44 | 3.08 | 0.023062 | 0.118062 | 0.2 | | 0.094430 | −5.12 |

**Supplementary Figures**

Western blot analysis of ALIX/PDCD6IP from exosomes derived from SW480-7 and total cell lysates of SW480-7. A series of blot images were captured with camera exposure times ranging from 1 minute to 5 minutes.

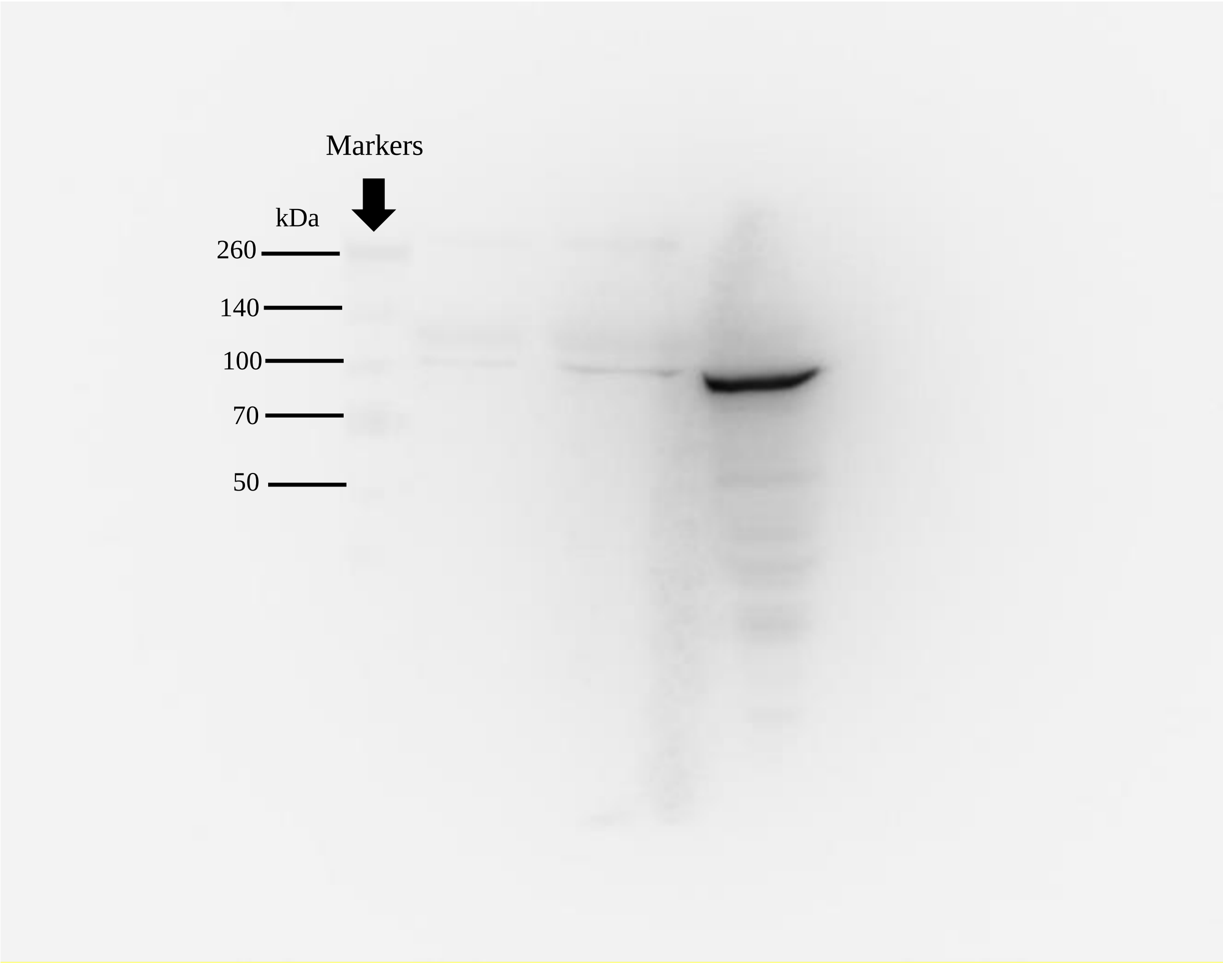


**Figure S1a.** Western blot analysis of ALIX/PDCD6IP from exosomes derived from SW480-7 and total cell lysates of SW480-7. The chemiluminescencec image was captured at 1 minute exposure time.

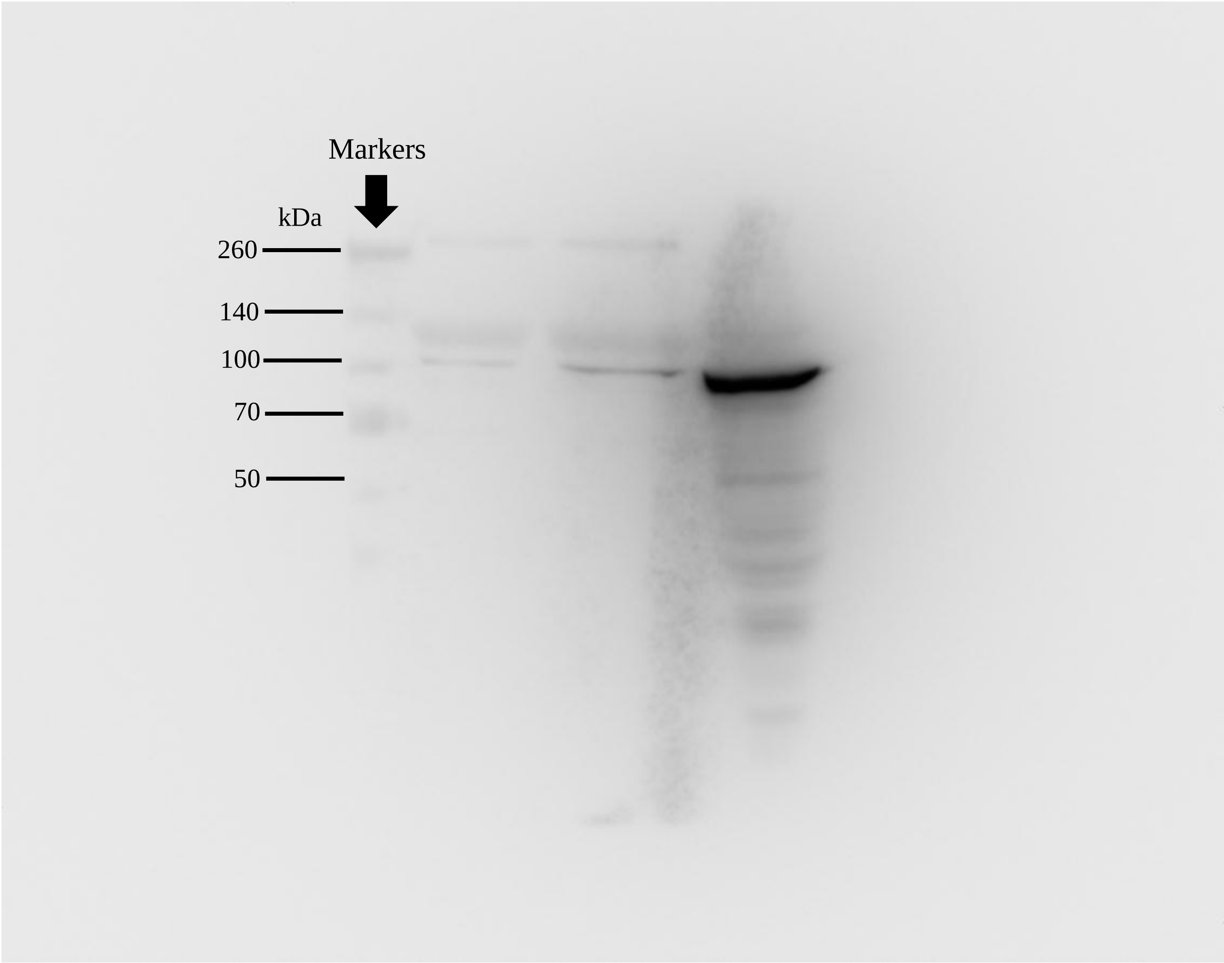


**Figure S1b.** Western blot analysis of ALIX/PDCD6IP from exosomes derived from SW480-7 and total cell lysates of SW480-7. The chemiluminescence image was captured at 2 minute exposure time.

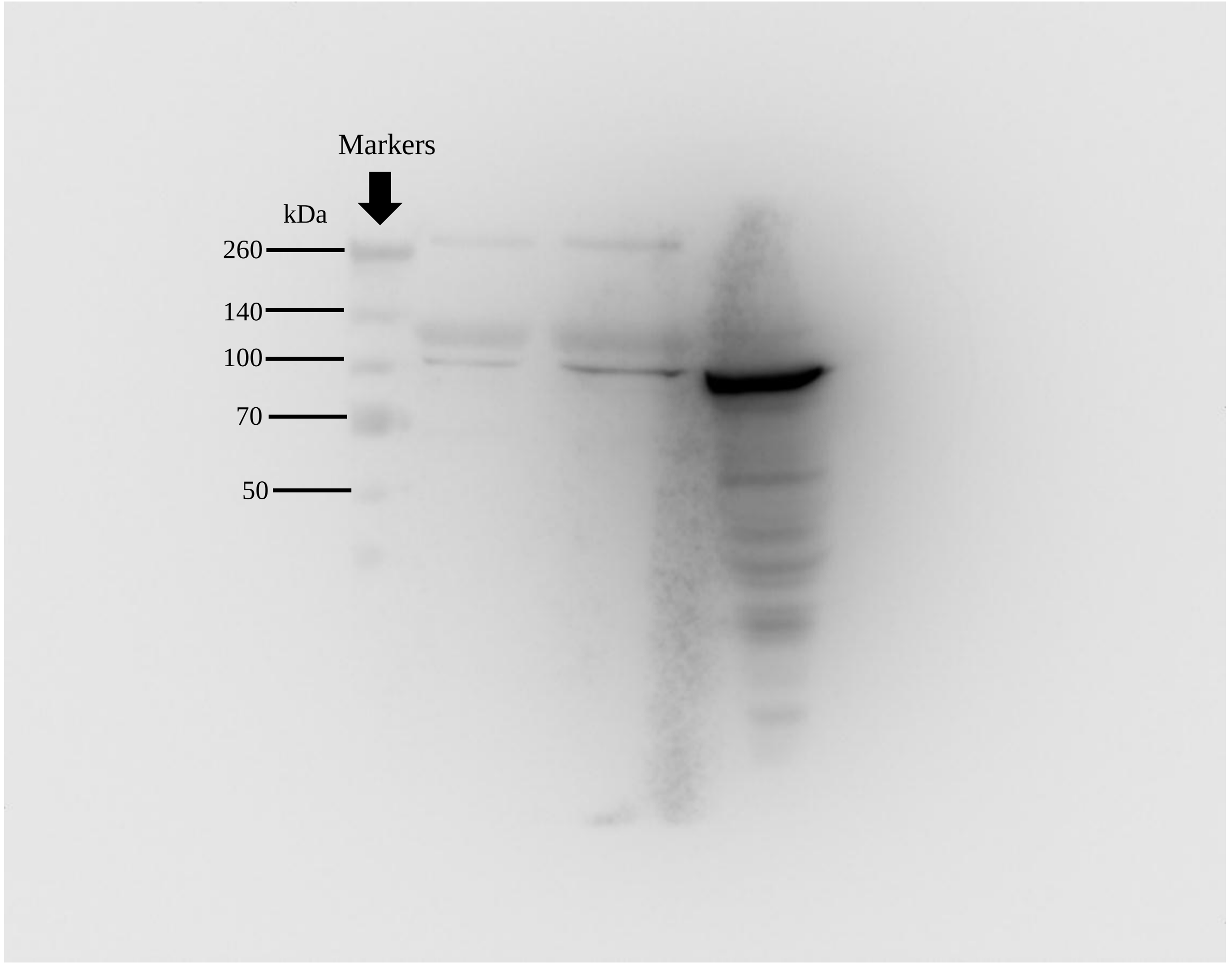


**Figure S1c.** Western blot analysis of ALIX/PDCD6IP from exosomes derived from SW480-7 and total cell lysates of SW480-7. The chemiluminescence image was captured at 3 minute exposure time.

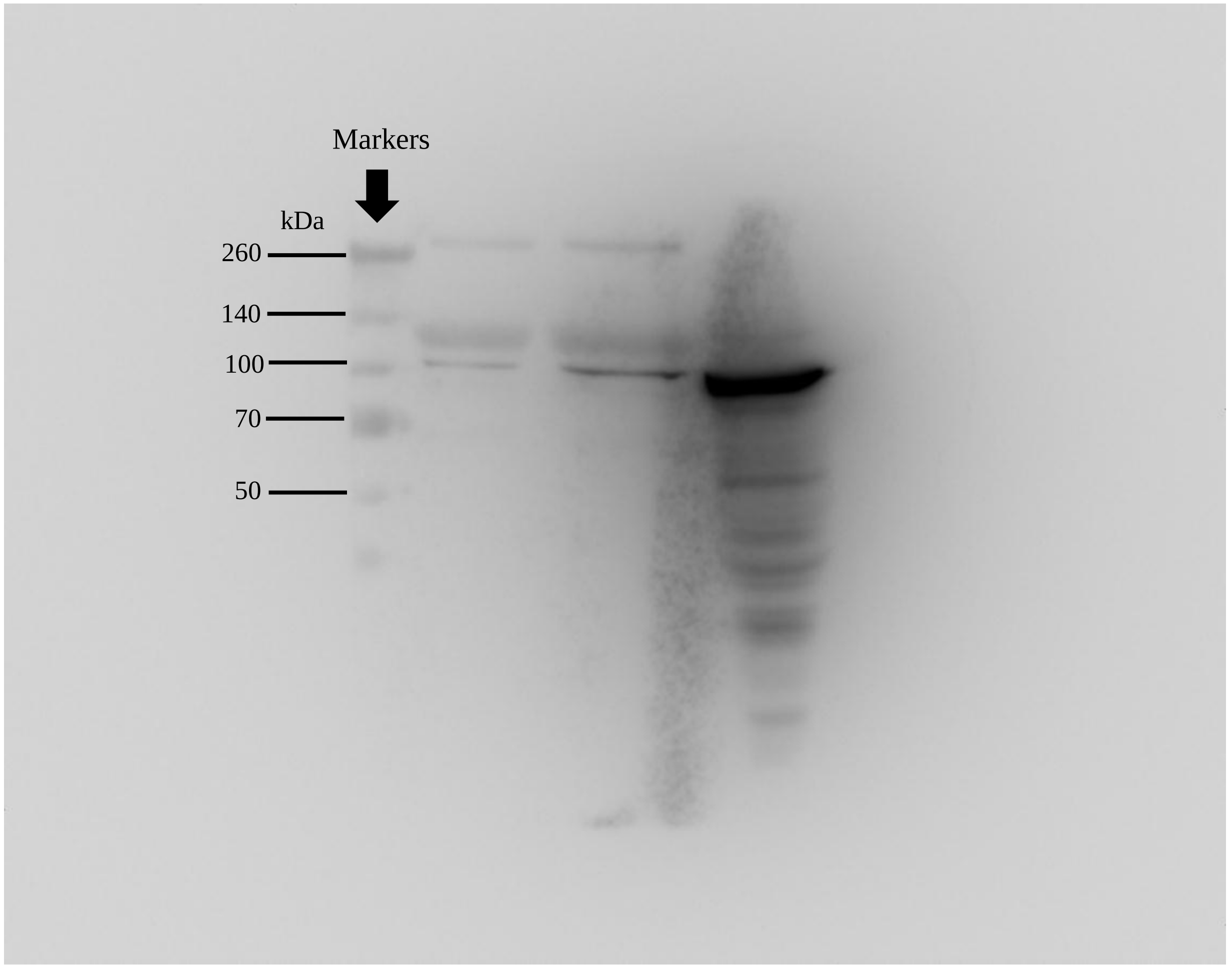


**Figure S1d.** Western blot analysis of ALIX/PDCD6IP from exosomes derived from SW480-7 and total cell lysates of SW480-7. The chemiluminescence image was captured at 4 minute exposure time.

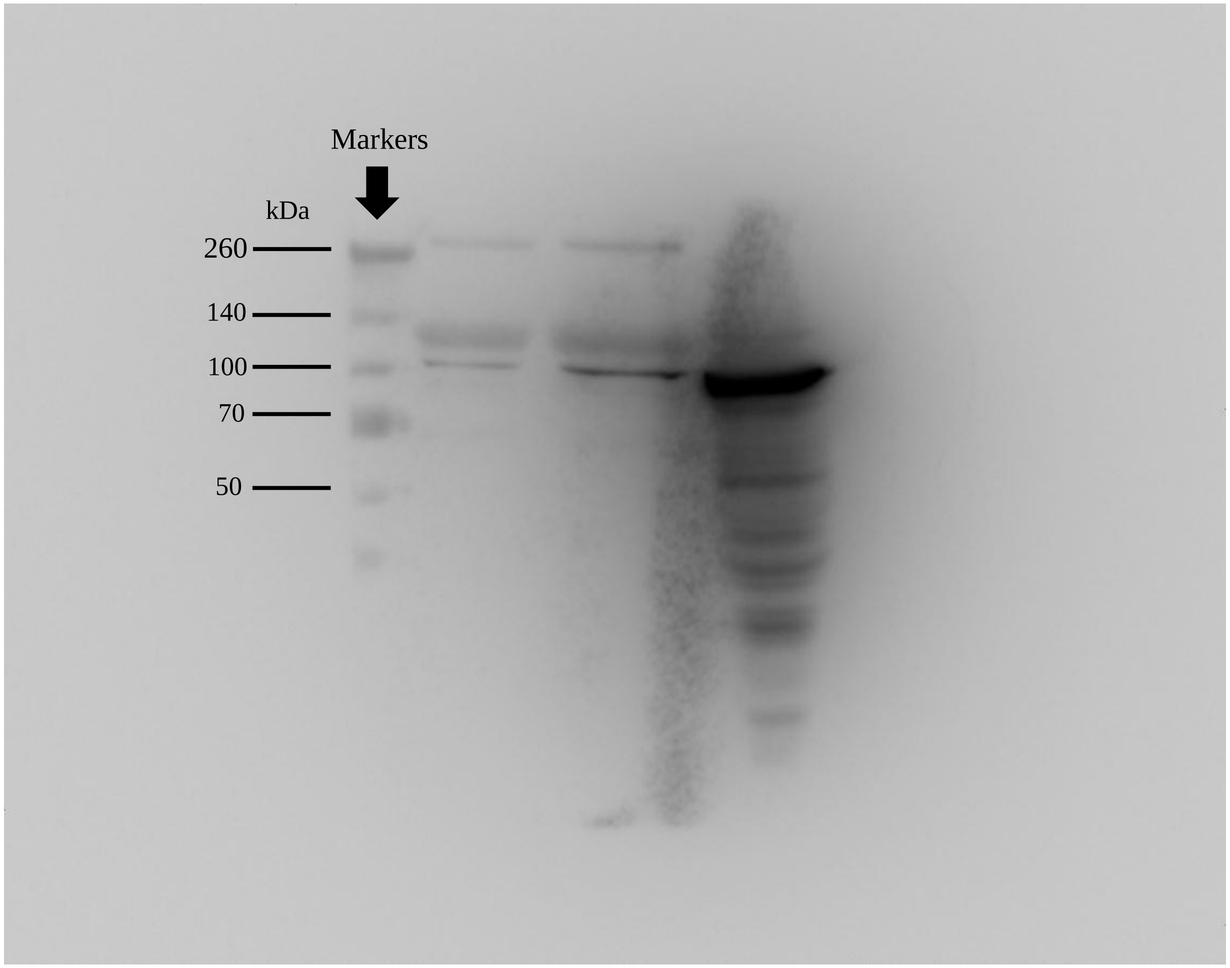


**Figure S1e.** Western blot analysis of ALIX/PDCD6IP from exosomes derived from SW480-7 and total cell lysates of SW480-7. The chemiluminescence image was captured at 5 minute exposure time.
